# Monitoring Drug and Antidrug Levels: A Rational Approach in Rheumatoid Arthritis Patients Treated with Biologic Agents Who Experience Inadequate Response While Being on a Stable Biologic Treatment

**DOI:** 10.1155/2014/702701

**Published:** 2014-05-20

**Authors:** Diana Mazilu, Daniela Opriş, Cecilia Gainaru, Mihaela Iliuta, Natalia Apetrei, Giorgiana Luca, Andreea Borangiu, Tania Gudu, Alexandra Peltea, Laura Groseanu, Cosmin Constantinescu, Ioana Saulescu, Violeta Bojinca, Andra Balanescu, Denisa Predeteanu, Ruxandra Ionescu

**Affiliations:** ^1^Department of Rheumatology and Internal Medicine, “Sfanta Maria” Hospital, Bulevardul Ion Mihalache nr 37-39, 011172 Bucharest, Romania; ^2^“Carol Davila” University of Medicine, Bucharest, Romania; ^3^Biology Faculty, Bucharest University, Bucharest, Romania; ^4^Biology Faculty, “Alexandru Ioan Cuza” University, Iasi, Romania

## Abstract

Clinical response in patients with rheumatoid arthritis (RA) treated with biologic agents can be influenced by their pharmacokinetics and immunogenicity. The present study evaluated the concordance between serum drug and antidrug levels as well as the clinical response in RA patients treated with biological agents who experience their first disease exacerbation while being on a stable biologic treatment. 154 RA patients treated with rituximab (RTX), infliximab (IFX), adalimumab (ADL), or etanercept (ETN) were included. DAS28, SDAI, and EULAR response were assessed at baseline and reevaluated at precise time intervals. At the time of their first sign of inadequate response, patients were tested for both serum drug level and antidrug antibodies level. At the next reevaluation, patients retreated with RTX that had detectable drug level had a better EULAR response (*P* = 0.038) with lower DAS28 and SDAI scores (*P* = 0.01 and *P* = 0.03). The same tendency was observed in patients treated with IFX and ETN regarding EULAR response (*P* = 0.002 and *P* = 0.023), DAS28 score (*P* = 0.002 and *P* = 0.003), and SDAI score (*P* = 0.001 and *P* = 0.026). Detectable biologic drug levels correlated with a better clinical response in patients experiencing their first RA inadequate response while being on a stable biologic treatment with RTX, IFX, and ETN.

## 1. Introduction

Rheumatoid arthritis (RA) is a chronic inflammatory disease that can result in substantial morbidity [1–3], impaired physical activity, and poor quality of life [4, 5], leading to a reduced life expectancy by 3 to 18 years [6] and increased mortality [7–11].

The targets of biologic agents are interactions between the immune cells (mainly T lymphocytes, B lymphocytes, and macrophages), which are responsible for inflammation and structural damage in affected joints, and the signaling molecules involved in their activation. The most used approved biologic agents for the treatment of RA are tumor necrosis factor (TNF) antagonists (infliximab, adalimumab, etanercept, golimumab, and certolizumab) or products that target B cells like rituximab (a chimeric monoclonal antibody that targets CD20 B cells) or inhibitor of costimulation of T cells (abatacept). Most of these agents are very effective at improving the signs and symptoms and at slowing or preventing structural damage in patients with RA [12–21]. Since the introduction of biologic treatment, prognosis of the disease has been substantially improved [22, 23].

Nevertheless, despite all these therapeutic advances and their relatively expensive costs, a variable proportion of patients with several autoimmune diseases including RA and inflammatory bowel diseases (IBD), who initially benefited from biologics, eventually lost response [24–26]. For example, a study from the Swedish TNF-antagonist registry found that 44% of patients were still taking their initial therapy at five years, and 25% were no longer taking any TNF antagonist at all [25]. As for IBD, up to 50% of patients lose response to treatment (secondary nonresponders) and up to 30% do not respond at all (primary nonresponders) [27]. The rational for lack or loss of response is multifactorial: molecular structure of biologic drug, pharmacokinetics, pharmacodynamics, and development of anti-drug antibodies.

In IBD, there are several strategies to the management of secondary failure to TNF antagonists [26]. These include switching to another drug in the same or different class, increasing the dose of biologic drug, changing concomitant immunosuppressive drug, or measuring drug levels and antibodies [28–30]. Therapeutic drug monitoring seems to be the adequate approach for the biologic treatment management [28]. Testing for drug levels and antibodies in secondary nonresponders is more cost-effective when compared to empiric drug escalation [31, 32]. It identifies those patients who will benefit from dose escalation versus those who are unlikely to respond to this strategy (high titers of anti-drug antibodies) [33].

Drug immunogenicity is one of the main mechanisms behind therapeutic failure also for RA patients [34–38]. Systemic reviews and meta-analysis conclude that anti-drug antibodies are clinically relevant and lead to significant decrease of therapeutic response [39]. Dose escalation in these patients may boost anti-drug antibodies production with serious adverse events [37, 40–42]. As for nonresponders without anti-drug antibodies but with detectable serum drug levels, these may respond better when switched to a drug with different mechanisms of action [43].

## 2. Methods

During a period of 2 years (January 2012–January 2014), we followed up 154 patients with established RA receiving one of the following biologic agents: rituximab (62 patients), infliximab (32 patients), etanercept (45 patients), and adalimumab (15 patients) with concomitant conventional synthetic disease-modifying antirheumatic drug (csDMARD) and few cases of monotherapy. Patients were included in order of their admission to the Department of Rheumatology, “Sfanta Maria” Hospital, Bucharest, Romania. All patients were previously diagnosed with RA according to ACR 1987 criteria [44] or ACR/EULAR 2010 criteria [45] and were treated using “treat to target” strategy [46] and local guidelines for the management of active RA [47]. The study was approved by the hospital Ethics Committee and all patients gave written informed consent before the study was started.

Demographic data, clinical (number of tender and swollen joints) and laboratory (ESR-erythrocyte sedimentation rate, CRP: C reactive protein, RF: rheumatoid factor, ACPA: anticyclic citrullinated peptide, IgG type) variables were collected at baseline and at each reevaluation. RA activity was evaluated in all patients by using 3 variables: Disease activity scores (DAS28 4v), Simplified Disease Activity Index (SDAI), and European League Against Rheumatism (EULAR) response. All clinical evaluations were performed by two independent assessors. As it was proposed at OMERACT 9 (Outcomes in Rheumatology) meeting [48], a RA flare represents a cluster of symptoms of sufficient duration intensity to require (re)initiation, change, or increase in therapy. Nevertheless, as suggested by several reports [49], in clinical research these criteria may be difficult to apply. Since there is no definition validated, we considered the situation as RA flare when at least one of the following conditions occurred: an increase in SDAI, an increase in ESR and/or CRP not due to a concomitant infection, an increase in DAS score to moderate or high disease activity, and a lower class in EULAR response as compared to previous reevaluation. At the moment of RA flare as described before, just before a new administration, patients were tested for anti-drug antibodies and biologic drug serum levels. According to serum drug levels patients were classified in group A if their serum drug levels were detectable and in group B if their drug levels were undetectable.

Patients were excluded from testing if their RA flare was related to conventional synthetic or biologic DMARD discontinuation, or a concomitant infectious disease, also if between baseline (the moment of serum drug level testing) and next reevaluation; patients had a change in their treatment regimen (increase in glucocorticoid dose and csDMARD dose or addition of a new immunosuppressive drugs). These particular patients were excluded from the final analysis. The reevaluation and clinical responses were assessed for each biologic drug: after 6 months from drug level testing, for RTX; after 2 months, just before a new i.v. infusion, for IFX; and after 3 to 4 months, for ETN and ADL.

### 2.1. Detection of Serum Drug Level and Anti-Drug Antibodies

Serum drug and antidrug levels were measured by enzyme linked immunosorbent assay (ELISA), using Progenika kits (Promonitor-RTX, Promonitor-anti-RTX, Promonitor-IFX, Promonitor-anti-IFX, Promonitor-ETN, Promonitor-anti-ETN, Promonitor-ADL, and Promonitor-anti-ADL). Several assays and technologies have been approved for monitoring serum drug and antidrug level [50], but bridging ELISA seems to be the only method with the potential for routine adoption in a hospital clinical setting for patient monitoring [37, 43, 51, 52]. It has been demonstrated that antibodies against TNF antagonists are anti-idiotypic, therefore neutralizing by definition [53]. Other technologies like cell-based assays, biacore, and homogeneous mobility shift assays can characterize the functionality of anti-drug antibodies; however, the question arises whether characterization of the antibody binding activity is required, when this can be easily answered with a simple ELISA test due to the fact that the immune response detected by ELISA is neutralizing. ELISA assays detect binding antibodies regardless of their functional activity. This method is similar for any other solid-phase methods like radioimmunoassays (RIA).

The clinical relevance of the immune response detected by ELISA is very well established and demonstrated in several studies [37, 51, 54, 55]. Promonitor kits have high accuracy for quantifying serum drug level, a pivotal importance to develop therapeutic algorithms [56].

In regards to drug levels detected by Promonitor kits, these span all clinically relevant drug concentrations (35–14400 ng/mL, 24–12000 ng/mL, 35–40000 ng/mL, and 665–240000 ng/mL for IFX, ADL, ETN, and RTX levels, resp.). ELISA tests used in this work have demonstrated an excellent correlation with other commercially available assays used for drug monitoring [56].

Cut-points of the anti-drug antibody tests are determined to be 2 AU/mL, 3.5 AU/mL, 142 AU/mL, and 340 AU/mL for anti-IFX, anti-ADL, anti-ETN, and anti-RTX antibodies, respectively. No human anti-drug antibody is currently available for anti-drug antibody screening; therefore outputs are given in arbitrary units per milliliter.

### 2.2. Statistical Analysis

Statistical analysis was performed using SPSS statistical software, version 20.0. The data were expressed as the mean ± SD. All statistical tests were two-sided and were performed at an *α* level of 0.05. The differences between groups were analyzed by Student's *t*-test, Kruskal-Wallis test, or Mann-Whitney test, as appropriate. Spearman's test was used for correlations.

## 3. Results

### 3.1. Characteristics of the Cohort

The study included 154 patients with established RA. One hundred and ten of them had a clinical or laboratory condition suggesting a disease flare during the evaluated period. Since final analysis, 38 patients met the exclusion criteria (8 patients had a significant increase in their glucocorticoid dose, 12 patients had csDMARD dose increase, 7 patients had a new csDMARD added to their treatment regimen, and 11 patients were switched to another biologic drug).

The final cohort of tested patients had the following treatment characteristics: 34.72% RTX patients (25 patients), 27.77% IFX patients (20 patients), 25% ETN patients (18 patients), and 12.5% ADL patients (9 patients). Their mean current biologic agent treatment was 41.79 ± 27.76 months in patients with RTX treatment, 34.45 ± 27.76 months with IFX, 49.38 ± 38.03 months with ETN, and 45.56 ± 23.88 months with ADL. The results showed that no detectable anti-drug antibodies were found in patients receiving RTX, ADL, and ETN. Patient's baseline characteristics are listed in Table 1.

At the moment of disease flare, 36% patients from the RTX group had undetectable drug level with 66.66% of them having moderate and high disease activity, mean DAS28 score of 3.45 ± 1.20. SDAI was lower in patients with detectable drug levels compared to patients with undetectable drug levels, 20.0 ± 15.7 versus 21.7 ± 29.6. There was no significant difference between groups A and B regarding both DAS28 score and SDAI (*P* = 0.678 and *P* = 0.845) nor treatment duration (27.75 ± 13.71 versus 48.81 ± 53.94, *P* = 0.294).

We found a significant difference in RTX serum level depending on ACPA status (*P* = 0.021). ACPA presence was positively associated with detectable RTX levels (OR = 8.75; 95%CI 1.21–63.4; *P* = 0.032) being a moderate predictor with AUC = 0.715; 95%CI: 0.5239–0.9067. This new finding supports the idea that patients positive for ACPA achieve a better clinical result being on treatment with B-cell depletion therapy. The mechanism by which these patients have higher RTX serum drug level should be studied further.

Interestingly, RTX serum level also correlated with the increased number of previous biologic agents (*P* = 0.009, *r* = 0.514). Sixty-two percent of patients with detectable serum RTX level had 2 anti-TNF agents as previous biologic treatment. Mention should be made that according to local guidelines, RTX is a second line biologic drug.

In the IFX treated patients, 90.90% (10 patients) of those with undetectable IFX serum level had moderate and high disease activity. Seven (63.63%) of these patients had anti-IFX antibodies. Surprisingly, anti-IFX antibodies were also found in 2 patients with subtherapeutic drug level. Twelve patients (60%) had a csDMARD associated: 8 patients had methotrexate, one patient had azathioprine, two patients had leflunomide, and one patient had sulfasalazine. Six patients did not have a csDMARD associated. Methotrexate dose range was between 7.5 mg and 20 mg/week. Our results showed that methotrexate association and the presence of anti-IFX antibodies were negatively correlated (*P* = 0.048, *r* = −0.447), confirming that methotrexate reduces IFX immunogenicity.

No anti-ETN antibodies were found in the 18 patients treated with ETN. At baseline, 77.77% of them had moderate and high disease activity evaluated by using DAS28 score and only 3 patients had undetectable drug levels. Also in this subgroup, there were 5 (27.7%) patients without a csDMARD, but all of them had detectable drug levels. Seven patients had methotrexate associated ranging from 10 mg to 20 mg/week and 6 patients had leflunomide 20 mg/day.

The group of patients treated with ADL that had a RA flare and were tested for drug levels was relatively small; 9 patients out of 15 patients enrolled in the study. Their mean DAS28 score was of 3.41. Moderate disease activity was found in 55.55% of them. No anti-ADL antibodies were detected. Only one patient had no csDMARD associated. Seven patients had methotrexate associated (10–20 mg/week, mean dose 15 mg/week) and one patient had leflunomide 20 mg/day.

### 3.2. Therapeutic Responses at Next Reevaluation after RA Exacerbation

During the follow-up period, patients from the final analysis remained on the same therapeutic treatment regimen regarding conventional synthetic and biologic DMARDs. Their EULAR responses are listed in Table 2.

Six months after testing the serum drug levels, RTX treated patients that had detectable drug levels at baseline (group A) and had a mean DAS28 2.93 ± 1.20 compared to 3.27 ± 1.47 in group B (*P* = 0.01). Twenty-two percent of patients from group B still had high disease activity according to DAS28 score and only 3 patients in this group obtained remission. The differences in disease activity (remission, low, moderate, and high) using DAS28 score were significant between groups A and B (*P* = 0.003). There was also a significant difference in their SDAI evolution: mean SDAI in group A was 12.23 ± 14.13 and in group B was 14.83 ± 20.51 (*P* = 0.033).

Regarding EULAR response (no, moderate, and good) in RTX treated patients there was a significant difference in the evolution of the two groups (*P* = 0.038). Twelve patients from group A achieved good and moderate response compared to only 3 patients from group B (Table 2).

All patients treated with IFX were reevaluated after 2 months. The difference in DAS28 evolution between group A and group B was significant: 3.67 ± 1.24 versus 5.59 ± 1.07 (*P* = 0.002). None of the patients having undetectable drug level at first RA flare achieved remission or low disease activity. Clinical response was also significantly different regarding also SDAI evolution (group A mean SDAI 17.26 ± 12.29 compared to group B mean SDAI 44.33 ± 18.22, *P* = 0.001). EULAR response was better in patients having detectable drug level at flare (*P* = 0.002) (Table 2).

Anti-drug antibodies were detected in 45% of IFX treated patients: seven patients (35%) had undetectable IFX level and 2 patients (10%) had subtherapeutic IFX level. All patients having anti-IFX antibodies had no EULAR response at follow-up and appropriate therapeutic management was initiated. Patient's characteristics are listed under positive and negative anti-IFX antibodies (Table 3).

At follow-up, higher DAS28 score was observed in patients with undetectable ETN levels compared to those from group A (7.17 ± 1.21 versus 3.57 ± 1.65, *P* = 0.003). Similar results were obtained in regards SDAI evolution: mean SDAI in group A was 19.06 versus mean SDAI in group B of 58.73 (*P* = 0.026). Patients with detectable ETN drug levels had better EULAR response (*P* = 0.023).

There was a relatively small number of patients treated with ADL. Mean DAS28 after 4 months of treatment from RA flare was 2.20 ± 0.38 in patients with detectable drug levels. Only one patient with undetectable drug level consequently had moderate disease activity at follow-up. No anti-ADL antibodies were found in patients treated with ADL.

## 4. Discussions

Current recommendation for the management of RA does not address serum biologic drug monitoring in clinical practice [46] even if biologicals possess a large pharmacokinetic variation. Thus, if a better disease control is aimed at measuring drug level seems appropriate [57].

RTX detectable drug level correlated with better clinical response at follow-up. We found a significant difference in RTX drug level at the moment of inadequate response in patients with positive and negative ACPA status. In a number of studies, serum concentration of ACPA and RF decreases during RTX treatment [58, 59], but their relation to RTX serum level has not been studied yet. As is known, there are biomarkers that seem to predict a good EULAR response: no steroid therapy, low lymphocyte count, and high RF level and BAFF levels [60]. Meanwhile, in larger observational cohort study, ACPA was a better biomarker of good EULAR response than RF [61]. Whether RF and/or ACPA positivity predict a better clinical response to RTX still remains to be demonstrated.

In our study, IFX serum drug level at the moment of inadequate response correlated with clinical activity. There was a significant difference in patient's EULAR response at follow-up; patients that had detectable serum drug levels had a better response. The presence of anti-IFX antibodies correlated to disease activity using DAS28 score at baseline; all of the patients with anti-drug antibodies had no EULAR response at follow-up. Methotrexate dose has an impact on INF immunogenicity and appropriate therapeutic approach should be made to reduce its immunogenicity.

As is well known, ETN has the lowest immunogenicity [62] and in our study none of the patients experiencing inadequate response had anti-ETA antibodies. Even though a proportion of them did not have a csDMARD associated there were no differences in serum drug levels. The data obtained in the ADL treated group was not significantly relevant because of the number of patients. But this cannot exclude the utility of serum drug and anti-drug dosing in patients treated with ADL.

Our results showed that evaluation of drug levels in patients that experience inadequate response while being on biologics correlate to their clinical response at follow-up. Thus it can be possible to determine loss of efficacy starting from the first RA exacerbation in patients with stable biologic treatment. This approach can be used in view of a better disease control and appropriate therapeutic decision.

We acknowledge that our study cannot fully demonstrate whether biologic drug dosing is predictive for clinical response and nonresponsiveness. Further studies are essential as this may be an argument for switching to another biologic drug in RA patients.

## 5. Conclusion

To our knowledge, this is the first study that evaluates biologic drug levels at first inadequate response and their relation to further clinical response in patients with RA. Our study strongly supports the idea that serum drug monitoring should be considered in clinical practice during long-term use of biologic agents. It adds some evidence that immunogenicity has an impact in clinical response in patients with anti-drug antibodies. Measuring drug level and assessing immunogenicity in a RA flare might help to optimize and personalize usage of biological therapies.

## Figures and Tables

**Table 1:** Patient's characteristics at the moment of dosing biologic drug level.

Biologic agent	Current biologic treatment, duration, and mean	DAS28 baseline, mean	SDAI baseline, mean	csDMARD associated, no (%)	ACPA positive, no (%)	RF positive, no (%)
RTX						
Group A	48.8 ± 53.4	3.65 ± 1.12	20.0 ± 15.7	15 (60%)	14 (56%)	16 (64%)
Group B	27.7 ± 13.7	3.45 ± 1.19	21.7 ± 29.6	8 (32%)	4 (16%)	7 (28%)
*P*	0.294	0.678	0.845	0.667	**0.021**	**0.049**
IFX						
Group A	40.6 ± 39.9	3.57 ± 1.25	15.2 ± 19.7	6 (30%)	4 (20%)	7 (35%)
Group B	29.3 ± 17.5	5.42 ± 1.19	43.2 ± 29.6	6 (30%)	3 (15%)	4 (20%)
*P*	0.379	**0.003**	**0.026**	0.582	0.515	0.064
ETN						
Group A	47.8 ± 38.5	4.14 ± 1.44	31.6 ± 31.3	10 (55.55%)	11 (61.11%)	12 (66.67%)
Group B	57.6 ± 23.7	5.25 ± 1.79	41.5 ± 40.3	3 (16.67%)	2 (11.11%)	2 (11.11%)
*P*	0.679	0.259	0.639	0.239	0.814	0.612
ADL						
Group A	46.7 ± 25.2	3.39 ± 1.04	10.1 ± 6.05	7 (77.78%)	4 (44.44%)	6 (66.67%)
Group B	36	3.54	32.9	1 (11.11%)	1 (11.11%)	1 (11.11%)
*P*	0.700	0.902	0.009	0.708	0.495	0.571

Differences between patient's baseline characteristics were tested by Student's *t*-test or chi-square test.

RTX: rituximab; IFX: infliximab; ETN: etanercept; ADL: adalimumab.

Group A: detectable drug level; Group B: undetectable drug level.

csDMARD: conventional synthetic disease modifying antirheumatic drug; ACPA: anticitrullinated peptides antibodies status; RF: rheumatoid factor status.

**Table 2:** EULAR responses at next reevaluation after first RA flare.

	EULAR response			
	No	Moderate	Good	*P*
RTX				
Group A	4 (16%)	5 (20%)	7 (28%)	**0.038**
Group B	6 (24%)	2 (10%)	1 (4%)	
IFX				
Group A	2 (10%)	5 (25%)	2 (10%)	**0.002**
Group B	10 (50%)	1 (5%)	0	
ETN				
Group A	3 (16.67%)	5 (27.78%)	7 (38.89%)	**0.023**
Group B	3 (16.67%)	0	0	
ADL				
Group A	2 (22.22%)	1 (11.11%)	5 (55.56%)	0.194
Group B	1 (11.11%)	0	0	

Differences between EULAR responses in group A and group B were tested using Kruskal-Wallis test, for each biologic agent.

RTX: rituximab; IFX: infliximab; ETN: etanercept; ADL: adalimumab.

Group A: detectable drug level; Group B: undetectable drug level.

**Table 3:** Patient's characteristics among positive and negative anti-IFX antibodies.

	Positive anti-IFX antibodies	Negative anti-IFX antibodies	*P* value
Disease duration, mean, and months	90.77 ± 49.56	128 ± 97.48	0.306
IFX treatment duration, mean, and months	29.77 ± 17.01	38.77 ± 34.60	0.511
DAS28 at flare and mean	5.09 ± 1.19	4.18 ± 1.67	0.189
DAS28 after 2 months and mean	5.68 ± 0.8	3.95 ± 1.49	**0.006**
csDMARD association and nr (%)	3	9	**0.028**

Differences between patient's characteristics were tested by Student's *t*-test or chi-square test.

IFX: infliximab; csDMARD: conventional synthetic disease modifying antirheumatic drug.
